# GILZ Modulates the Recruitment of Monocytes/Macrophages Endowed with a Resolving Phenotype and Favors Resolution of *Escherichia coli* Infection

**DOI:** 10.3390/cells12101403

**Published:** 2023-05-17

**Authors:** Laís C. Grossi, Isabella Zaidan, Jéssica Amanda Marques Souza, Antônio Felipe S. Carvalho, Rodrigo C. O. Sanches, Camila Cardoso, Edvaldo S. Lara, Ana Clara M. Montuori-Andrade, Stefano Bruscoli, Maria Cristina Marchetti, Carlo Riccardi, Mauro M. Teixeira, Luciana P. Tavares, Juliana P. Vago, Lirlândia P. Sousa

**Affiliations:** 1 Signaling in Inflammation Lab., Departamento de Análises Clínicas e Toxicológicas, Faculdade de Farmácia, Universidade Federal de Minas Gerais, Belo Horizonte 31270-901, Brazil; laiscgf@gmail.com (L.C.G.); bellazaidanmoreira@gmail.com (I.Z.); jessicaamanda.biomed@gmail.com (J.A.M.S.); afs.carvalho@hotmail.com (A.F.S.C.); camila.faceg@gmail.com (C.C.); souzaedvaldopht@gmail.com (E.S.L.); anaclaramatoso@gmail.com (A.C.M.M.-A.); 2 Programa de Pós-Graduação em Ciências Farmacêuticas, Faculdade de Farmácia, Universidade Federal de Minas Gerais, Belo Horizonte 31270-901, Brazil; 3 Hospital das Clínicas da Universidade Federal de Minas Gerais/Ebserh, Belo Horizonte 30130-100, Brazil; 4 Departamento de Bioquímica e Imunologia, Instituto de Ciências Biológicas, Universidade Federal de Minas Gerais, Belo Horizonte 31270-901, Brazil; rcosanches@gmail.com; 5 Department of Medicine and Surgery, Section of Pharmacology, University of Perugia, 06132 Perugia, Italy; stefano.bruscoli@unipg.it (S.B.); maria.marchetti@unipg.it (M.C.M.); carlo.riccardi@unipg.it (C.R.); 6 Centro de Pesquisa e Desenvolvimento de Fármacos, Departamento de Bioquímica e Imunologia, Instituto de Ciências Biológicas, Universidade Federal de Minas Gerais, Belo Horizonte 31270-901, Brazil; mmtex.ufmg@gmail.com; 7 Pulmonary and Critical Care Medicine Division, Department of Medicine, Brigham and Women’s Hospital and Harvard Medical School, Boston, MA 02115, USA; lpaduatavares@bwh.harvard.edu; 8 Experimental Rheumatology, Department of Rheumatology, Radboud Institute for Molecular Life Sciences, Radboud University Medical Center, 6525 GA Nijmegen, The Netherlands; julypri@gmail.com

**Keywords:** GILZ, mononuclear cells, migration, resolution of inflammation, *Escherichia coli*

## Abstract

Macrophages are important effectors of inflammation resolution that contribute to the elimination of pathogens and apoptotic cells and restoration of homeostasis. Pre-clinical studies have evidenced the anti-inflammatory and pro-resolving actions of GILZ (glucocorticoid-induced leucine zipper). Here, we evaluated the role of GILZ on the migration of mononuclear cells under nonphlogistic conditions and *Escherichia coli-*evoked peritonitis. TAT-GILZ (a cell-permeable GILZ-fusion protein) injection into the pleural cavity of mice induced monocyte/macrophage influx alongside increased CCL2, IL-10 and TGF-β levels. TAT-GILZ-recruited macrophages showed a regulatory phenotype, exhibiting increased expression of CD206 and YM1. During the resolving phase of *E. coli*-induced peritonitis, marked by an increased recruitment of mononuclear cells, lower numbers of these cells and CCL2 levels were found in the peritoneal cavity of GILZ-deficient mice (GILZ^−/−^) when compared to WT. In addition, GILZ^−/−^ showed higher bacterial loads, lower apoptosis/efferocytosis counts and a lower number of macrophages with pro-resolving phenotypes. TAT-GILZ accelerated resolution of *E. coli-*evoked neutrophilic inflammation, which was associated with increased peritoneal numbers of monocytes/macrophages, enhanced apoptosis/efferocytosis counts and bacterial clearance through phagocytosis. Taken together, we provided evidence that GILZ modulates macrophage migration with a regulatory phenotype, inducing bacterial clearance and accelerating the resolution of peritonitis induced by *E. coli*.

## 1. Introduction

Leukocyte recruitment during inflammation is a tightly coordinated process that ensures a proper immune response, clearance of the insult and mediates the repair response sites [1]. Different cellular effectors mark the phases of inflammation, from the onset to resolution [2]. Elucidating the mechanisms involved in each of the phases informs therapeutic opportunities for inflammatory diseases [3]. Of interest, the nonphlogistic recruitment of monocytes/macrophages during the resolution phase of inflammation promotes the elimination of apoptotic neutrophils via efferocytosis, preventing secondary necrosis of these cells and promoting resolution of the inflammatory process [4].

The resolution of inflammation is marked by an active recruitment of macrophages, which is crucial for the clearance of debris, microorganisms and apoptotic cells and further tissue repair [1,2,4]. Macrophages adopt different phenotypes depending on environmental cues [1]. Regulatory/pro-resolving macrophage phenotypes feedforward the resolution process by secreting pro-resolving molecules that further contribute to enhancing the functions of these cells [4]. The recruitment of monocytes is mainly dependent on the chemokine CCL2. Noteworthily, it has been reported that pro-resolving mediators are able to recruit monocytes/macrophages with a regulatory phenotype dependent on CCL2 and on its receptor CCR2 [4].

The glucocorticoid-induced leucine zipper (GILZ) is a protein induced by glucocorticoids and has key roles in the control of the immune response [5,6]. The role of GILZ in different inflammatory diseases has been explored in pre-clinical studies that highlighted the potent anti-inflammatory and pro-resolving actions of this mediator [5,6]. The exogenous administration of GILZ leads to the inhibition of pro-inflammatory pathways and their mediators (IL-6 and TNF-α) [6], the induction of neutrophil apoptosis [7] and reprogramming of macrophages to resolve phenotypes [8]. In addition, recent studies suggest that GILZ also plays an important role in promoting bacterial clearance during experimental polymicrobial sepsis [9,10] and pneumococcal pneumonia [11], resulting in decreased tissue damage and increased survival rates [9,10,11].

Given the substantial expression of GILZ in regulatory macrophages and its actions in the resolution of inflammation and infection [5,6,7,8,9], the hypothesis tested here was that TAT-GILZ promotes the nonphlogistic recruitment of mononuclear cells, skewing the response towards an M2/pro-resolving pattern. Thus, here, we evaluated the mechanisms for TAT-GILZ in the resolution of *E. coli*-induced peritonitis.

## 2. Materials and Methods

### 2.1. Mice

Male C57BL/6 mice (8–10 weeks old) were obtained from the central animal facility of the Universidade Federal de Minas Gerais. Mice had free access to commercial chow and water. GILZ-deficient male mice (GILZ^−/−^) and their C57BL/6 WT littermates were a generous donation from Prof Eric Morand (Monash University, Clayton, Australia). Mice were bred in the animal facility of the Immunopharmacology Laboratory and generated as described [12]. The GILZ gene is X-chromosome-linked, and male GILZ^−/−^ mice are infertile [13,14]. Therefore, females GILZ^+/−^ are crossed with GILZ^+/+^ males, generating about 20–25% of male knockout mice in every breeding cycle. All procedures described were approved by the local animal ethics committee (CEUA 183/2017).

### 2.2. Bacteria

*Escherichia coli* (ATCC 25922) was grown in Miller Hinton (MH) medium for 18 h/37 °C. Subsequently, bacterial colonies were transferred to 15 mL of LB broth (Luria Bertani) and incubated at 37 °C for 12 h. Bacterial inoculum was prepared in sterile saline. In all experiments, the inoculum was confirmed by spreading bacterial suspension in MacConkey agar plates.

### 2.3. Cell Culture—BMDMs and Peritoneal Macrophages

Wild-type C57BL/6 mice and GILZ^−/−^ were euthanized, and the bone marrow was collected from tibias and femurs. Cells were then centrifuged for 5 min at 1200× *g*. The pellet was resuspended in a suitable media for BMDM differentiation (RPMI with 10% heat-inactivated fetal bovine serum plus 30% L929 cell-conditioned medium), seeded on flasks and incubated at 37 °C and 5% CO_2_. After 7 days, the supernatant was collected, and adherent macrophages were detached by using a cell scraper and plated (2 × 10^5^ cells/well) in 96-well plates for phagocytosis assay or seeded in 24-well plates (1 × 10^6^ cells/well) to evaluate cytokine level.

To obtain peritoneal macrophages, male C57BL/6 WT and GILZ^−/−^ mice were submitted to peritoneal lavage to harvest the macrophages present in the cavity. The process was performed aseptically and in a laminar flow cabinet. Cells were collected with 3 mL of sterile PBS using a Pasteur pipette and gently poured into 15 mL tubes. Subsequently, cell suspension was centrifuged at 1300 RPM for 5 min and resuspended in 1 mL of PBS/3% BSA. A 20 µL aliquot of the suspension was taken and diluted 1:10 in Turk solution for cell counting. Further, 2.6 × 10^5^ cells were plated per well in 96-well plates and subjected to phagocytosis assay 2 h after macrophage adherence.

### 2.4. TAT-GILZ Source and Purity

The TAT peptide and the TAT-GILZ fusion protein, constructed by inserting GILZ cDNA into the TAT-C vector to produce an in-frame fusion protein, were generated as previously described [15]. Each batch of TAT and TAT-GILZ was produced using a modified strain of *E. coli* BL21 bacteria (ClearColi BL21), producing a modified and non-immunogenic LPS and minimizing the possibility of an endotoxic response in immune cells [11,16]. In addition, any presence of residual LPS after protein purification was screened to detect possible endotoxin (LPS) traces, as described [7]. Proteins were dissolved in DMSO and diluted further in sterile saline. TAT and TAT-GILZ dosages were chosen according to previously published studies [7,8,11]. Equimolar doses of TAT and TAT-GILZ were injected, considering that the molecular weight (MW) of TAT is half of TAT-GILZ.

### 2.5. In Vitro Production of Cytokines

BMDMs from WT C57BL/6 mice were washed with serum-free RPMI and subsequently incubated with TAT (1 µg/mL), TAT-GILZ (2 µg/mL) or RPMI (untreated), as previously described [11]. At 6, 12, 24 and 48 h after treatment, the supernatant was collected for IL-10, TGF-β and CCL2 evaluation via ELISA. The cell extract was used for Western blot analysis.

### 2.6. Cell Migration Models

A cohort of mice received an intrapleural (i.p.) injection of TAT-GILZ (100 ng/cavity), TAT (50 ng/cavity) or sterile saline solution and they were euthanized at different intervals (4, 12, 24 and 48 h). Pleural lavage was performed to collect the recruited leukocytes. Flow cytometry was employed to validate microscopic findings at 48 h post-injection. Pharmacological inhibition of CCR2 was achieved through use of the antagonist RS504393 (2 mg/kg, i.p.) from Tocris Bioscience (Ellisville, MO, USA) 1 h before TAT-GILZ injection [4]. Pelleted cells were also used for Western blot analysis. CCL2 levels were measured via ELISA at different time points post-TAT-GILZ injection.

In the self-resolving model of peritonitis, WT and GILZ^−/−^ mice received an injection into the peritoneal cavity with 1 × 10^6^ CFU of *E. coli* [4]. At 6, 24 and 48 h post-infection, mice were euthanized, and peritoneal leukocytes were harvested for total and differential cell counts. The percentage of cells with highly distinctive apoptotic morphology and efferocytosis (macrophages that ingested apoptotic cells) was obtained by counting cytospin slides stained with May–Grunwald–Giemsa (500 cells/slide were counted) [11,17,18,19]. CCL2 levels were measured in cell-free supernatants from peritoneal lavages using ELISA. Bacterial loads were determined by seeding the peritoneal lavage harvested at 6 and 48 h post-infection in MacConkey agar (USB Corporation, Cleveland, OH, USA) plates and incubated overnight at 37 °C. In some experiments, C57BL/6 mice received treatment with TAT-GILZ (5 µg/cavity), TAT (2.5 µg/cavity) or vehicle 12 h post-infection (hpi). Exudates were collected within 24 h for total and differential leukocyte count, bacterial load and evaluation of neutrophil apoptosis [19] and efferocytosis events [4,8,11,17,18].

### 2.7. Flow Cytometry

Leukocytes were stained with the following antibodies: F4/80 (PE–clone BM8, eBioscience, San Diego, CA, USA), Ly6C (PeCy7–clone HK1.4, eBioscience, San Diego, CA, USA), Ly6G (BV421–clone 1A8, BioLegend, San Diego, CA, USA), CD3 (Alexa 488–clone 17A2, BioLegend), CD11b (BV-500–clone M1/70, Pharmingen, San Jose, CA, USA) and CD206 (APC–clone C068C2, BioLegend). Populations of macrophages (F4/80^+^), monocytes (Ly6C^+^/F4/80^−^), neutrophils (Ly6G^+^) and lymphocytes (CD3^+^) were assessed. Events were acquired in FACSCanto II (BD Biosciences, Franklin Lakes, NJ, USA) and analyzed using FlowJo Software (Tree Star Inc., Ashland, OR, USA). Macrophage phenotypes were defined based on the expression of F4/80, CD11b, Ly6C and CD206 [4,17,20].

### 2.8. ELISA

Levels of IL-10, TGF-β, CCL2, CXCL1, TNF-α and IL-6 were measured in the supernatants obtained from pleural and peritoneal lavages. ELISA was performed using commercially available antibodies according to the procedures supplied by the manufacturer (R&D Systems, Minneapolis, MN, USA).

### 2.9. Western Blotting

Western blot analyses were performed as previously described [18,21]. Samples were resolved on denaturing 10% polyacrylamide SDS gels followed by transfer to nitrocellulose membranes. Membranes were blocked for 1 h in PBS (5% non-fat dry milk; 0.1% Tween-20) and incubated overnight with antibodies anti-Ym1 (#60130, 1:1000—StemCell Technologies, Vancouver, BC, Canada), anti-P-STAT3 (#9134, 1:1000–Cell Signaling Technology, Beverly, MA, USA) or anti–β-actin (1:10,000, #A5316–AC-74, Sigma-Aldrich, St. Louis, MO, USA). Secondary anti-rabbit (#7074, Cell Signaling Technology) or anti-mouse (#sc-2005, Santa Cruz Biotechnology, Dallas, TX, USA) HRP-conjugated antibodies (1:3000) were added to the membranes and incubated for 1 h at room temperature. ECL detection system (GE Healthcare, Chicago, IL, USA) was used to visualize immunoreactive bands. Autoradiographs were scanned, and densitometry analysis was conducted in ImageJ software (Bethesda, MA, USA). Results were expressed as arbitrary units and normalized using β-actin as loading controls.

### 2.10. Phagocytosis Assays

Phagocytosis was evaluated as previously described [4,11,20]. Briefly, 2 × 10^5^ peritoneal cells or BMDMs isolated from WT and GILZ^−/−^ mice were plated and incubated with *E. coli* (MOI of 10) for 3 h to allow for phagocytosis (1 h of adhesion at 4 °C followed by 2 h at 37 °C and 5% CO_2_). Noninternalized bacteria were incubated with gentamicin (abx) (5 µg/mL in PBS) for 30 min. In order to evaluate phagocytosed bacteria, macrophages were lysed as described [4,11,20] and plated in MacConkey agar plates (overnight at 37 °C and 5% CO_2_) to count viable bacteria. Eighteen hours before the experiments, a pre-treatment with TAT or TAT-GILZ was conducted in doses based on previous studies [11]. Data were expressed as graphs of internalized bacteria (CFU count) or percentage of phagocytosis. To obtain the percentage of phagocytosis, the average CFU of the WT mice was calculated (considered as 100%), and the individual values of each animal were achieved by comparing to 100%.

### 2.11. Statistical Analysis

Statistical analysis was carried out through analysis of variance (two-way ANOVA) followed by Bonferroni’s multiple comparisons test or one-way ANOVA, followed by the Newman–Keuls post-test to compare more than two groups. Differences were considered statistically significant if *p* < 0.05. Some results required unpaired *t*-test for the comparison of two groups. Outliers were excluded from the analyses. Results were presented as mean ± SEM (standard error of the mean). Statistical analyses and graphs were prepared using the GraphPadPrism Software 6.0 (San Diego, CA, USA).

## 3. Results

### 3.1. TAT-GILZ Induces a Time-Dependent Migration of Mononuclear Cells Accompanied by Increased Levels of CCL2

**Figure 1:**
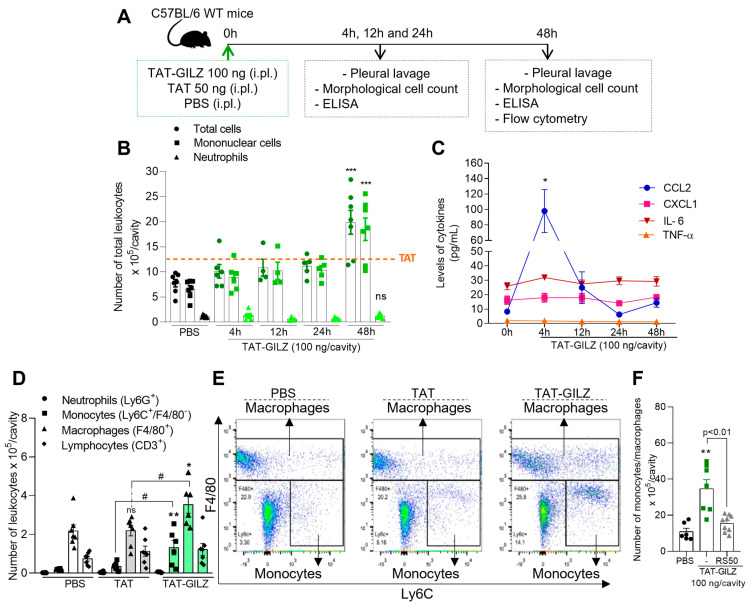
Time-dependent monocyte/macrophage recruitment to the pleural cavity after TAT-GILZ injection. C57BL/6 mice received an intrapleural (i.pl.) injection of TAT (50 ng), TAT-GILZ (100 ng) or PBS (control); (**A**) experimental design, and (**B**) cells recruited into the cavity were harvested at 4, 12, 24 and 48 h for total and differential leukocyte counts via light microscopy of slides stained with May–Grunwald–Giemsa. (**C**) Levels of IL-6, TNF-α, CXCL1 and CCL2 were measured using ELISA in pleural lavage supernatants. Flow cytometry analysis of recruited leukocytes collected 48 h after TAT, TAT-GILZ or PBS injection was also performed. (**D**) Leukocyte expressed as absolute numbers and (**E**) flow cytometry gating strategy. (**F**) Recruited monocytes/macrophages into cavity after pre-treatment with the CCR2 antagonist RS504393 (2 mg/kg, i.p.) (*n* = 6–9). Results are presented as mean ± SEM (graphs **B**,**C**, *n* = 5–7; **D**, *n* = 6–7). * denotes *p* < 0.05, ** *p* < 0.01 and *** *p* < 0.001 when compared to control (PBS); # denotes *p* < 0.05 when compared to control TAT, via one-way ANOVA.

In order to evaluate whether GILZ could promote leukocyte recruitment, we first performed in vitro chemotaxis assays using murine macrophages and human neutrophils. We found that TAT-GILZ, but not TAT, promoted macrophage chemotaxis rather than neutrophils, and enhanced the levels of CCL2 produced by bone-marrow-derived macrophages (BMDMs) (Supplementary Figure S1A–C). To validate these findings in in vivo settings, cell recruitment kinetics was evaluated by injecting TAT-GILZ or their respective control, TAT, into the pleural cavity of mice (Figure 1A). TAT-GILZ injection promoted a significant leukocyte influx into the pleural cavity of mice at 48 h after injection (Figure 1B), which was mostly composed of mononuclear cells. Noteworthily, TAT-GILZ-induced migration of mononuclear cells was accompanied by increased levels of the chemokine CCL2, peaking at 4 h, without affecting CXCL1 levels and the pro-inflammatory cytokines IL-6 and TNF-α (Figure 1C). Immunophenotyping of recruited cells revealed that mononuclear cells were the most prevalent leukocyte population upon TAT-GILZ injection (Figure 1D,E), with insignificant numbers of neutrophils at all time points analyzed. Of note, as seen in in vitro settings, pleural injection of TAT did not promote leukocyte migration to the pleural cavity and also did not modify the levels of chemokines and cytokines (available in Supplementary Figure S2A,B and depicted as an orange line in Figure 1A).

Given that TAT-GILZ was able to induce CCL2 release in vitro (Supplementary Figure S1C) and in vivo (Figure 1C), we next investigated the relevance of the pathway engaged by CCL2 and its receptor CCR2 [22] for TAT-GILZ-induced monocyte/macrophage recruitment. For that, we carried out experiments giving the CCR2 antagonist RS504393 (2 mg/kg, i.p.) [4,17] prior to TAT-GILZ injection. We found that pre-treatment in mice with the CCR2 antagonist reduced the recruitment of monocytes/macrophages to the pleural cavity at the 48 h time point (Figure 1F). Taken together, these findings show that TAT-GILZ induced a nonphlogistic recruitment of monocytes/macrophages via CCR2.

### 3.2. TAT-GILZ Promotes Macrophage Polarization towards Regulatory Phenotypes

**Figure 2:**
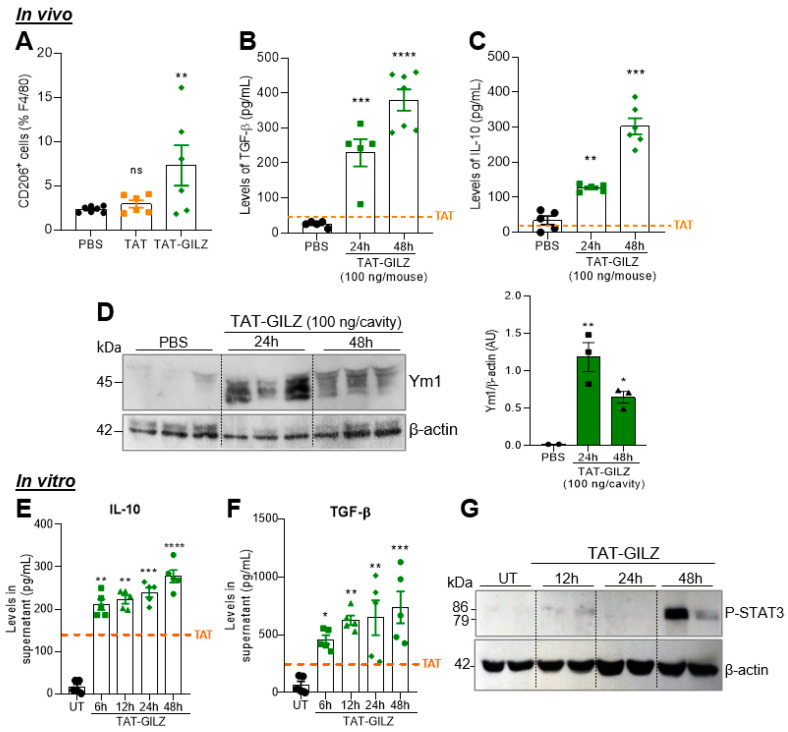
Evaluation of M2 markers after TAT-GILZ injection or BMDM stimulation. C57BL/6 mice received an i.pl. injection of TAT (50 ng), TAT-GILZ (100 ng) or PBS (control), and pleural washes were harvested at 24 and 48 h. Flow cytometry (A) was performed at 48 h time point. Western blot analysis in whole cell extracts and ELISA for IL-10 and TGF-β in supernatants were performed at two time points post-injection. In vitro experiments were carried out using BMDMs stimulated with TAT (1 µg/mL) or TAT-GILZ (2 µg/mL). (**A**) Number of macrophages recruited to the cavity that were positive for the CD206 marker. (**B**,**C**) IL-10 and TGF-β levels in pleural lavage supernatants of mice injected with TAT-GILZ at different time points. (**D**) Western blot analysis of Ym1 in cells harvested of pleural cavity and accompanying densitometry. β-Actin was used as a loading control. Kinetics of (**E**) IL-10 and (**F**) TGF-β production by BMDMs in cell-free supernatants. (**G**) Representative blot from two different experiments for P-STAT3 using whole extracts from TAT-GILZ-stimulated BMDMs (*n* = 2). In vivo data are presented as mean ± SEM of 5–7 mice per group. Results in graphs E-F are representative of 2 independent experiments with BMDMs performed in biological quintuplicates. Western blot quantification was performed using ImageJ software. * Denotes *p* < 0.05, ** *p* < 0.01, *** *p* < 0.001 and **** *p* < 0.0001 when compared with the control group (PBS), via one-way AN0VA.

Next, we evaluated the phenotypic profile of macrophages into the pleural cavity after TAT-GILZ injection. Interestingly, TAT-GILZ increased the frequency of macrophages positive for CD206, a classical M2 marker [23] (Figure 2A), and the secretion of the regulatory cytokines IL-10 and TGF-β (Figure 2B,C). Confirming the macrophage phenotype, increased Ym1 expression (M2 marker) was detected in cells harvested from pleural cavity at 24 and 48 h post-TAT-GILZ injection (Figure 2D and accompanying densitometry). Consistent with our in vivo findings, in vitro assays showed that TAT-GILZ induced IL-10 and TGF-β secretion via BMDMs (Figure 2E,F), associated with increased STAT3 phosphorylation (Figure 2G), an important signaling pathway involved in the induction of the M2 phenotype [18,24,25,26,27]. Of note, although TAT alone did not influence the levels of IL-10 and TGF-β in vivo (Figure 2B,C), it had some effect on these cytokines levels in BMDMs but at lower levels than those induced by TAT-GILZ (Figure 2E,F), see threshold levels in orange lines of graphs 2B,C and 2E,F. Taken together, our data show that TAT-GILZ promoted the migration of monocytes/macrophages to the pleural cavity of mice and induced a regulatory phenotype of macrophages.

### 3.3. Endogenous GILZ Is Important for Mononuclear Cell Recruitment, Bacterial Clearance and Efferocytosis in a Self-Resolving Model of Peritonitis Induced by E. coli

**Figure 3:**
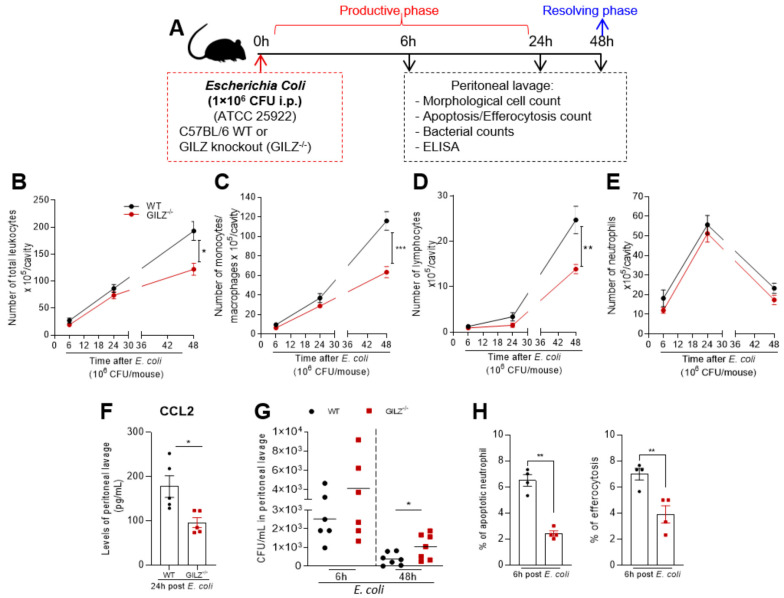
Effect of GILZ absence in a self-resolving model of peritonitis. WT and GILZ^−/−^ mice were infected with *E. coli* (1 × 10^6^ CFU, i.p.), and cells were harvested from peritoneal cavity at 6, 24 and 48 h after infection; (**A**) experimental design. Numbers of (**B**) total leukocytes, (**C**) monocytes/macrophages, (**D**) lymphocytes and (**E**) neutrophils were evaluated at different time points via morphological counts of cytospin slides stained with May–Grunwald–Giemsa. (**F**) CCL2 levels were measured in cell-free supernatants from peritoneal washes 24 h after infection. (**G**) CFU numbers in peritoneal washes at 6 and 48 h after infection (*n* = 6–7). (**H**) The percentage of apoptotic neutrophils and efferocytosis was evaluated by counting cytospin slides. Data are presented as mean ± SEM, * denotes *p* < 0.05, ** *p* < 0.01 and *** *p* < 0.001, via two-way ANOVA (**B**–**E**) or *t* test when comparing 2 groups (**F**–**H**).

Given the functional relevance of GILZ in monocyte/macrophage migration and polarization, our next step was to evaluate the endogenous role of this protein in leukocyte migration in a self-resolving model of peritonitis induced by *E. coli*. In this model of acute inflammation, the resolution phase occurs spontaneously within 48 h post-infection, based on the clearance of neutrophils from the inflamed cavity, and is accompanied by increased mononuclear cell numbers [4,28]. First, the temporal recruitment of leukocyte infiltration into the peritoneum of wild-type (WT) littermates and GILZ-deficient mice (GILZ^−/−^) was analyzed after *E. coli* challenge (scheme in Figure 3A). Increased migration of leukocytes to the peritoneal cavity was observed after *E. coli* infection, with similar kinetics in both genotypes. Nevertheless, GILZ^−/−^ mice exhibited lower leukocyte recruitment at the resolution time point (i.e., 48 h post-infection; Figure 3B). Of interest, the decreased recruitment of cells observed was related to a reduction in monocyte/macrophage and lymphocyte counts in the peritoneal cavity of GILZ^−/−^ (Figure 3C,D). No differences between genotypes were detected in the neutrophil numbers post-infection (Figure 3E). The reduced number of mononuclear cells in GILZ^−/−^ mice was accompanied by lower levels of CCL2 (Figure 3F), higher bacterial counts (Figure 3G) and lower percentages of apoptotic neutrophils and efferocytosis (Figure 3H), when compared to WT mice.

### 3.4. The Lack of Endogenous GILZ Results in Lower Number of Regulatory Macrophages during E. coli Peritonitis

**Figure 4:**
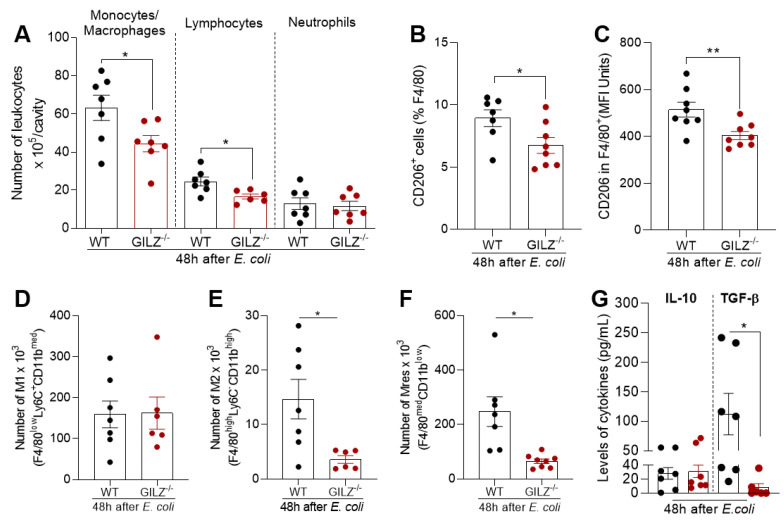
Effect of GILZ deficiency on macrophage phenotype in *E. coli*-induced peritonitis model. Flow cytometry analysis of leukocyte population recruited to the peritoneal cavity of the WT and GILZ^−/−^ mice was performed 48 h after *E. coli* infection. (**A**) Representation of the absolute numbers of monocytes (F4/80^−^/Ly6C^+^)/macrophages (F4/80^+^/CD11b^+^), lymphocytes (CD3^+^) and neutrophils (F4/80^−^/Ly6G^+^). (**B**) Frequency and (**C**) mean fluorescence intensity (MFI) of CD206^+^ in macrophage population. Subpopulations of macrophages (**D**–**F**) classified as M1 (F4/80^low^/Ly6C^+^/CD11b^med^), M2 (F4/80^high^/Ly6C^−^/CD11b^high^) and Mres (F4/80^med^/CD11b^low^) were expressed in absolute numbers. (**G**) IL-10 and TGF-β levels evaluated in supernatants from peritoneal cavity washes. Results are shown as the mean ± SEM (*n* = 7). * Denotes *p* < 0.05, ** *p* < 0.01 when compared GILZ^−/−^ vs. WT mice, via *t* test.

To better characterize the leukocytes recruited in both genotypes during the resolving phase of *E. coli*-induced peritonitis, flow cytometry experiments were performed using cell surface markers for macrophages (F4/80), neutrophils (Ly6G), monocytes (Ly6C) and lymphocytes (CD3) [4,17]. In line with the morphological counts of leukocytes (shown in Figure 3E), the deficiency in GILZ did not impact the number of neutrophils (Ly6G^+^) recruited to the cavity of mice during this self-resolving model of inflammation. On the other hand, populations of monocytes/macrophages and lymphocytes were decreased in 48 h infected GILZ^−/−^ compared to WT littermates (Figure 4A). Further immunophenotyping of the recruited macrophages showed a reduced expression of CD206 in the cells of GILZ^−/−^ mice (Figure 4B,C). To explore the macrophage phenotype more comprehensively, we applied a gating strategy that allows for the evaluation of different macrophage phenotypes based on surface marker expression [4,17,18]. During the resolving phase of *E. coli*-induced peritonitis, the number of classically activated macrophages (F4/80^low^/Ly6C^+^/CD11b^med^) did not alter in GILZ-deficient mice (Figure 4D), but the amount of M2 (F4/80^high^/Ly6C^−^/CD11b^high^) and Mres (F4/80^med^/CD11b^low^) macrophages was lower in GILZ^−/−^ mice compared to their respective WT littermates (Figure 4E,F). In keeping with that, lower levels of TGF-β were detected in the peritoneal cavity of GILZ^−/−^ mice, without changes in IL-10 levels (Figure 4G). These data obtained from the pre-clinical model of *E. coli*-evoked peritonitis reinforce and add to those from LPS-induced pleurisy [8], pointing out GILZ as an important molecule involved in macrophage reprogramming toward regulatory phenotypes in different inflammatory settings.

### 3.5. TAT-GILZ Enhances Resolution of Peritonitis induced by E. coli

**Figure 5:**
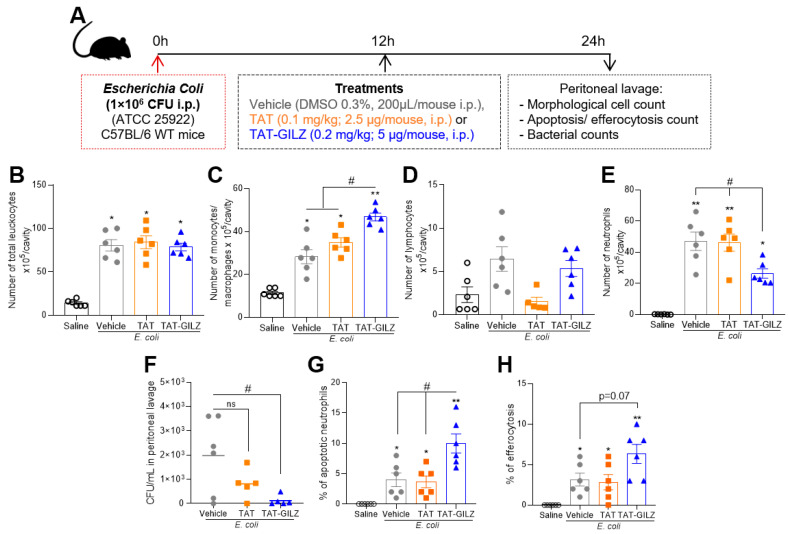
Effect of TAT-GILZ treatment in *E. coli*-induced peritonitis. C57BL/6 mice were infected with *E. coli* (1 × 10^6^ CFU, i.p.) and then treated with TAT (0.1 mg/kg, i.p.) or TAT-GILZ (0.2 mg/kg, i.p.) 12 post-infection. Mice were euthanized 24 hpi. (**A**) Schematic protocol. (**B**) Numbers of total leukocytes, (**C**) monocytes/macrophages, (**D**) lymphocytes and (**E**) neutrophils into peritoneal cavity evaluated via morphological counts of cytospin slides stained with May–Grunwald–Giemsa. (**F**) Bacterial counts in peritoneal lavage. Percentage of apoptotic neutrophils (**G**,**H**) efferocytosis. Data are mean ± SEM (*n* = 6 mice/group). * Denotes *p* < 0.05 and ** *p* < 0.01 when compared to the saline injected group, or as indicated by # *p* < 0.05, when comparing TAT-GILZ-treated peritonitis to vehicle or TAT groups, via one-way ANOVA.

Based on the endogenous relevance of GILZ during *E. coli*-induced peritonitis shown in Figure 4, we sought to understand the potential therapeutic effect of TAT-GILZ in this model. Therefore, WT mice were challenged with *E. coli* (10^6^ CFU), treated 12 h post-infection with TAT-GILZ, TAT or PBS and samples harvested at the 24 h time point for evaluation of leukocyte infiltration, neutrophil apoptosis/efferocytosis and bacterial load (experimental design in Figure 5A). Despite the similar number of total leukocytes recruited to the peritoneal cavity observed in the infected groups (Figure 5B), the treatment of mice with TAT-GILZ increased the number of monocytes/macrophages at 24 h post-infection (Figure 5C), with no effect on lymphocyte numbers (Figure 5D) when compared to TAT or vehicle-treated mice. On the other hand, a decrease was reported for neutrophil numbers (Figure 5E). Moreover, TAT-GILZ treatment decreased the bacterial load (Figure 5F) and increased the percentage of apoptotic neutrophils (Figure 5G) and efferocytosis (Figure 5H) within the peritoneal cavity. Taken together, TAT-GILZ treatment of *E. coli*-induced peritonitis promoted key regulatory events that favored the resolution of inflammation and infection at 24 h post-infection.

### 3.6. GILZ Deficiency Impairs Macrophage Phagocytosis that Can Be Restored through Pre-Treatment with TAT-GILZ

**Figure 6:**
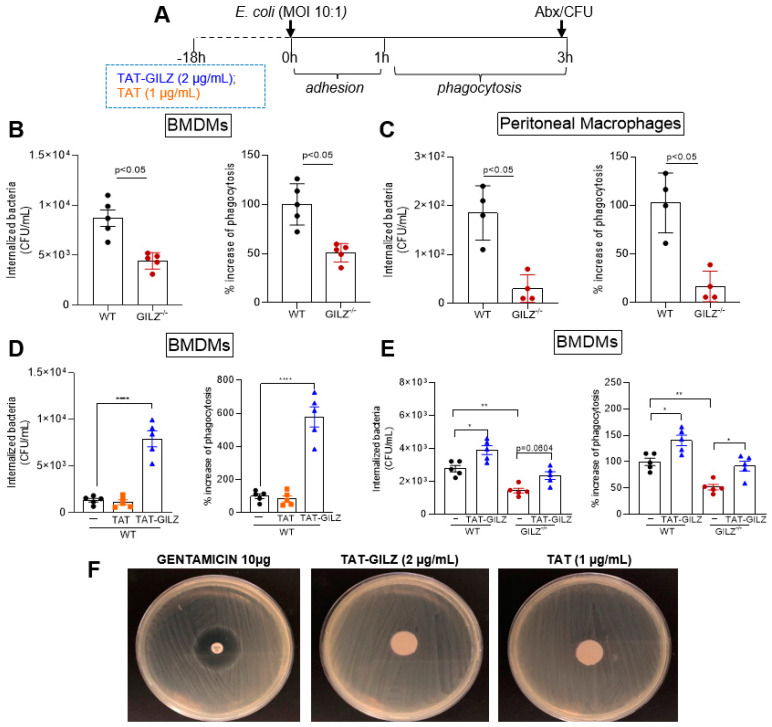
Role of GILZ on *E. coli* phagocytosis. Bacterial phagocytosis, experimental design in (**A**), was evaluated in (**B**) bone-marrow-derived macrophages (BMDMs) or (**C**) peritoneal macrophages obtained from WT and GILZ^−/−^ mice. (**D**) Macrophages from WT mice (2 × 10^5^ cells/well) were pretreated with TAT (1 μg/mL) or TAT-GILZ (2 μg/mL) for 18 h and then incubated with opsonized *E. coli* (MOI of 10) for 3 h to allow for adhesion and further phagocytosis. Non-internalized bacteria were removed by incubating the cells with gentamicin (Abx), followed by macrophage lysis and plating on MacConkey agar to identify the number of viable phagocytosed bacteria. In another experimental group, (**E**) BMDMs from GILZ^−/−^ mice (2 × 10^5^ cells/well) were also subjected to phagocytosis as described above. Results are expressed as CFU of internalized bacteria or % phagocytosis (CFU counts on McC agar plates, *n* = 4–5) as stated in M and M section and are presented as mean ± SEM; * denotes *p* < 0.05, ** *p* < 0.01 and **** *p* < 0.0001 when comparing cells treated with TAT-GILZ vrs vehicle or TAT, by 1-way ANOVA (**D**,**E**). The comparison between WT and GILZ^−/−^ BMDMs was performed using *t* test (* *p* < 0.05). Data are representative of 3 independent experiments performed in biological quadruplicates or quintuplicates. (**F**) TAT-GILZ (2 μg/mL), TAT (1 μg/mL) or gentamicin (control) impregnated on sterile filters were added to Mueller–Hinton agar plates containing *E. coli*. The growth inhibition zone was evaluated after incubation for 18 h at 37 °C.

Since GILZ^−/−^ mice had higher bacterial counts in peritoneal lavage post-infection, we wondered whether this was related to the reduced number of monocytes/macrophages, impaired function of these cells or both. For that, we used BMDMs and peritoneal macrophages from GILZ^−/−^ or WT mice to perform *E. coli* phagocytosis assays (see Methods and Figure 6A). Interestingly, in both macrophage types, GILZ deficiency led to a significantly lower number of internalized bacteria and consequent lower percentage of phagocytosis in comparison to WT macrophages (Figure 6B,C).

We next evaluated whether TAT-GILZ could impact *E. coli* phagocytosis via macrophages. To address that question, BMDMs from WT mice were treated with TAT (control 1 µg/mL) or TAT-GILZ (2 µg/mL) before the phagocytosis assay. Pre-treatment with TAT-GILZ promoted increased phagocytosis of bacteria when compared to vehicle- or TAT-treated cells (Figure 6D). We also wondered whether TAT-GILZ would be able to restore the phagocytic function of GILZ-deficient macrophages. To do so, the same protocol described above was applied. Interestingly, TAT-GILZ was able to restore phagocytic function of macrophages from GILZ^−/−^ mice to similar values of those found in WT macrophages (Figure 6E). To rule out a possible anti-bacterial effect of GILZ, paper discs impregnated with TAT, TAT-GILZ or the antibiotic gentamicin (10 μg, control) were added to Mueller–Hinton (MH) agar plates containing *E. coli*, and the growth zone inhibition was evaluated post-incubation. As expected, the positive control, gentamicin, showed an inhibition halo for *E. coli*, while TAT or TAT-GILZ, at the concentrations used in the assay, did not lead to bacterial inhibition, indicating that TAT-GILZ has no direct effect on bacterial growth (Figure 6F).

## 4. Discussion

Inflammation resolution is a complex process that restores tissue homeostasis through various mechanisms [29,30,31]. Endogenous pro-resolving mediators promote resolution by reducing pro-inflammatory mediators, promoting apoptosis of granulocytes and regulating macrophage recruitment and polarization [32]. By understanding the different phases of inflammation, therapeutic strategies can be developed to manage inflammatory diseases.

**Figure 7:**
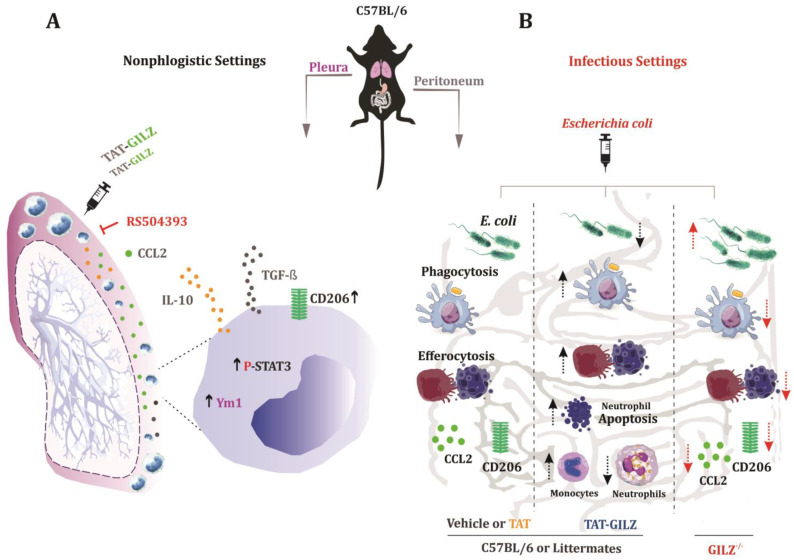
Schematic representation of the role of GILZ in monocyte/macrophage recruitment, reprogramming of macrophage and *E. coli* clearance from the peritoneum of mice. (**A**) The injection of TAT-GILZ in the pleural cavity of C57BL/6 mice induced CCL2/CCR2-dependent migration of monocytes/macrophages presenting resolving/regulatory phenotype. These cells showed increased CD206 and Ym1 expression, enhanced release of anti-inflammatory cytokines (IL-10 and TGF-β) and elevated levels of P-STAT3. (**B**) In the context of peritonitis induced by *E. coli*, GILZ deficiency was associated with lower monocyte/macrophage numbers into peritoneum, higher bacterial counts, lower neutrophil apoptosis and efferocytosis by macrophages, as well as lower expression of CD206 and CCL2 levels, when compared to their WT littermate controls. TAT-GILZ treatment of *E. coli-*infected mice decreased the neutrophil population in the inflammatory focus and increased the apoptosis of these cells. TAT-GILZ also promoted an increased influx of monocytes/macrophages into the peritoneum, which was associated with increased phagocytosis/efferocytosis and reduced bacterial counts into the cavity. Experiments performed in vitro revealed that GILZ increased the ability of macrophages to phagocytose *E. coli.* Up arrows denote increased effects and down arrows decreased effects.

In this study, we presented evidence for a major role of GILZ mediating the macrophage responses, especially during infection. More specifically, we showed that TAT-GILZ: (i) Increased the CCL2/CCR2-dependent monocyte/macrophage recruitment. (ii) Promoted macrophage polarization towards regulatory phenotypes. (iii) The deficiency of GILZ impaired the recruitment and altered the phenotype of macrophages during *E. coli* infection resolution. The deficiency of GILZ was associated with lower levels of CCL2, decreased apoptosis/efferocytosis and higher bacterial loads. Moreover, (iv) TAT-GILZ given at the peak of *E. coli*-evoked peritonitis promoted resolution by decreasing neutrophils and increasing monocyte/macrophage numbers, apoptosis/efferocytosis and bacterial clearance. Mechanistically, (v) GILZ improved macrophage phagocytosis of *E. coli*, with no direct bactericidal effect (summarized in Figure 7).

Pro-resolving molecules can include the essential fatty-acid-derived specialized pro-resolving mediators [33] and peptides and proteins, such as plasmin [20,34,35], annexin A1 [19,21,33,36,37] and angiotensin(1–7) [4,38]. More recently, the anti-inflammatory and pro-resolving properties of GILZ have been highlighted in different pre-clinical models of inflammatory diseases [5,7,8,11,15,39,40,41,42]. For instance, in a self-resolving model of LPS-induced pleurisy, GILZ expression was shown to be increased during the resolving phase of inflammation. Notably, the administration of TAT-GILZ, at the peak of inflammation, decreased the number of viable neutrophils by inducing apoptosis of this cells [7]. Furthermore, it was later reported that, in addition to promoting neutrophil apoptosis [7,43], GILZ induces other key events during the resolution phase of inflammation, including macrophage reprogramming and efferocytosis [8], pointing out GILZ as part of an endogenous resolution program [5]. Yet, the mechanisms underlying GILZ actions remain to be completely explored.

Growing evidence suggest that molecules with pro-resolving properties can induce migration of monocytes/macrophages in a nonphlogistic manner, especially through the CCL2/CCR2 axis [4,17,34]. Indeed, activation of the CCL2/CCR2 axis can promote the polarization of human and murine macrophages towards regulatory phenotypes [44,45]. Furthermore, CCL2/CCR2 is a canonical monocyte chemoattractant pathway [22] and enhances the removal of apoptotic cells by macrophages [46,47,48]. Monocytes/macrophages play a pivotal role during the immune response, and they are endowed with high phenotype plasticity, depending on the microenvironmental cues [49]. The expression pattern of surface markers can dictate cellular contrasting functions related to different cell phenotypes [50,51,52]. Here, TAT-GILZ injection promoted a rapid secretion of CCL2, increasing the nonphlogistic recruitment of monocytes, associated with the phosphorylation of STAT3 and the release of IL-10 and TGF-β. It is well established that regulatory/resolving macrophages release anti-inflammatory cytokines, such as IL-10 and very low levels of pro-inflammatory mediators [46,53]. Additional signatures of this phenotype, such as Ym1 (a member of the chitinase family) and CD206 (mannose receptor), were also identified [54,55]. Indeed, increased IL-10 produced by macrophages was associated with P-STAT3 activation and efferocytosis during inflammation resolution [56]. Macrophages with these profiles are important players in the resolution of the inflammatory response by promoting phagocytosis of bacteria/debris and efferocytosis of apoptotic granulocytes, mediating regenerative tissue responses [4,23,57].

During inflammation, the early recruited monocytes/macrophages are endowed with activated phenotypes that release pro-inflammatory mediators, in order to promote inflammation and eliminate the insulting agent more effectively [58]. These macrophages are didactically classified as M1 (classically activated macrophages). Once the harmful stimulus is neutralized, a switch in production of pro-inflammatory mediators to pro-resolving ones is observed, contributing to the functional transition of macrophages to a regulatory phenotype (M2 or alternatively activated macrophages) [59]. M2 macrophages are highly efferocytic and secrete anti-inflammatory cytokines, including IL-10 and TGF-β [57,59,60,61]. In addition, during the resolving phase of inflammation, there is a significant increase in the macrophage population inside the affected site [17]. In this sense, a distinct population of macrophages that appears in the inflammatory site during the resolution phase of inflammation was identified in vivo and named Mres or pro-resolving macrophages [57,59]. GILZ is remarkably expressed in M2 and Mres macrophages, observed during pleurisy resolution [7] and in vitro settings. Of note, no increase in GILZ levels is observed after stimulation with the M1-polarizing agents, such as LPS/IFN-γ [8]. These findings can be associated with the fact that activation of the glucocorticoid receptors leads to an increased expression of GILZ, which, in turn, inhibits transcription factors involved in pro-inflammatory pathways associated to M1 profile, such as NF-κB and AP1 [41,62,63,64]. It is worth mentioning that skewing the M1 macrophages to M2 is a highly dynamic process and can be reversed under physiological and pathological conditions [50]. In the course of various pathophysiological situations, the same signaling pathway may be involved in the polarization of M1 or M2 macrophages dependent on the phase of inflammatory response and the microenvironment cues [65]. Furthermore, an imbalance in signaling transduction pathways, as that involved in M1 toward M2 macrophage polarization, is associated with several chronic inflammatory diseases, from either infectious or non-infectious origin [66].

GILZ has drawn attention as a potential means of dissociating the beneficial anti-inflammatory effects of glucocorticoids from their deleterious effects [67]. Several studies have analyzed its protective aspect in colitis, in which transgenic mice that overexpress GILZ present lower levels of colonic inflammation [15,39]. The presence of GILZ decreased disease markers, demonstrating a protective action against severe inflammation [15]. In animal models of collagen-induced arthritis, GILZ silencing was associated with disease exacerbation, whereas mice overexpressing the gene had significantly less severe arthritis, both clinically and histologically [12,68]. Moreover, the use of the TAT-GILZ fusion protein has been shown to effectively attenuate inflammation in non-infectious inflammatory models, such as colitis [15,16], pleurisy [7,8] and kidney injury [69]. However, these studies have mostly focused on GILZ anti-inflammatory rather than pro-resolving actions, especially with regard to macrophage recruitment and function.

The exploitation of the pro-resolving activities of GILZ in infectious diseases is promising although still poorly described. In both a polymicrobial model of sepsis induced by CLP (cecal ligation and perforation) [10] and endotoxemia induced by high LPS dose [9]**,** the overexpression of GILZ in the macrophage lineage reduced systemic inflammatory cytokine levels and led to better survival, associated with reduced bacteremia and increased bacteria phagocytosis [9,10]. Indeed, transient overexpression of GILZ renders mice protected from LPS-induced endotoxemia [70]. Of interest, GILZ expression was lower in mice during endotoxemia and in septic patients [10,11]. Recently, we have shown that, in a model of acute lung injury (ALI), GILZ-deficient mice presented more severe damage, with excessive inflammation and decreased efferocytosis when compared to WT littermates [11]. Notably, treatment with TAT-GILZ reduced neutrophilic inflammation and prevented infection-associated lung damage in *Streptococcus pneumoniae*-induced ALI in mice. In this regard, TAT-GILZ increased macrophage counts in the airways, with a consequent improvement in efferocytosis and pneumococcus clearance [11]. Here, we showed that TAT-GILZ also affords protection during peritonitis induced by the Gram-negative bacteria *E. coli*, promoting the resolution of neutrophilic inflammation and improving bacterial clearance. Of interest, TAT-GILZ reduces leukocyte counts during inflammation by triggering cell death mechanisms [7,43]. Indeed, TAT-GILZ induces neutrophil apoptosis by deactivating key survival pathways on neutrophil, such as MCL-1 [7]. These data are consistent with the effect of GILZ in restraining neutrophil activation during a dinitrobenzene sulfonic acid (DNBS)-induced colitis model, in which the absence of GILZ resulted in an excessive inflammatory state with higher neutrophil numbers in lamina propria [42].

Here, GILZ deficiency did not affect neutrophil migration. This is not surprising since, during pleurisy-induced inflammation, GILZ^−/−^ mice have higher levels of AnxA1 compensating resolution responses and reducing neutrophil counts [7]. Akin to our data, Ricci et al. (2019) showed that GILZ deficiency does not impact the number of neutrophils after intraperitoneal infection with *Candida albicans* [42]. Yet, the authors observed higher neutrophil activation and yeast clearance in GILZ^−/−^ mice. Here, despite the same number of neutrophils after peritonitis with *E. coli*, we observed lower numbers of monocytes/macrophages associated with a greater number of bacteria in GILZ^−/−^ mice. These contrasting results might be related to differences in the infectious agent (bacteria vs. yeast). Most importantly, administration of TAT-GILZ to infected mice promoted resolution of neutrophilic inflammation and increased monocyte/macrophage population. These results were associated with higher percentages of apoptotic neutrophils, increased efferocytosis by macrophages and lower bacterial loads when compared to vehicle-treated mice. In agreement with our data, increased numbers of CD45^+^ leukocytes performing phagocytosis were found in peritoneal exudates of GILZ-overexpressing transgenic mice subjected to cecal ligation and puncture [10]. These findings suggest that endogenous GILZ modulates the inflammatory response, and its exogenous administration (through TAT-GILZ) favors inflammation resolution. One can hypothesize that the adoptive transfer of regulatory macrophages (induced by TAT-GILZ) might promote the resolution of *E. coli*-evoked peritonitis. Further studies are necessary to test this possibility.

In the model of *E. coli*-induced peritonitis, endogenous GILZ seems to be important for bacterial clearance, since GILZ deficiency leads to a greater number of bacteria in the peritoneal cavity, even when leukocyte numbers are similar in both WT and GILZ^−/−^. Additionally, pre-treatment of macrophages with TAT-GILZ increased the phagocytic potential of these cells and rescued this function in macrophages from GILZ-deficient mice. Indeed, overexpressing GILZ in the macrophage linage showed lower pro-inflammatory levels associated with increased survival rates during endotoxemia. In addition, those mice show increased phagocytosis of bacteria [9]. These results are in contrast to those showing that downregulation of GILZ increases phagocytosis of *S. typhimurium* by macrophages [71]. Overall, these studies indicate that the GILZ-mediated effects on phagocytosis vary according to the type of phagocytic cell and the bacteria strain employed. In agreement with the data obtained from a sepsis model [9,10], treatment of pneumococcal pneumonia with TAT-GILZ significantly decreased the bacterial load in the airways of mice. In the same study, it was shown that bacterial phagocytosis was impaired in the absence of GILZ, whereas treatment of WT macrophages with TAT-GILZ increased this function [11]. These findings are supported by those found in the present study, showing that GILZ deficiency interfered with *E. coli* phagocytosis in two macrophage types (bone-marrow-derived and peritoneal). Notably, the pre-treatment of macrophages with TAT-GILZ increased the phagocytic potential of these cells, as well as rescuing this function in macrophages from GILZ-deficient mice.

In summary, here, we uncovered a novel pro-resolving action for GILZ, promoting macrophage recruitment and enhancing anti-bacterial functions. This study highlights the crucial role for GILZ as a protective molecule in *E. coli-*induced peritonitis by regulating inflammation and promoting host macrophage responses to control bacteria.

## Data Availability

Data sets generated are available in the current manuscript.

## Supplementary Materials

The following supporting information can be downloaded at: https://www.mdpi.com/article/10.3390/cells12101403/s1, Figure S1: TAT-GILZ induces macrophage migration and CCL2 production in vitro; Figure S2: Effects of TAT injection into the pleural cavity of mice.

## References

[1] Murray P.J., Wynn T.A. (2011). Protective and pathogenic functions of macrophage subsets. Nat. Rev. Immunol..

[2] Alessandri A.L., Sousa L., Lucas C., Rossi A.G., Pinho V., Teixeira M.M. (2013). Resolution of inflammation: Mechanisms and opportunity for drug development. Pharmacol. Ther..

[3] Perretti M., Leroy X., Bland E.J., Montero-Melendez T. (2015). Resolution Pharmacology: Opportunities for Therapeutic Innovation in Inflammation. Trends Pharmacol. Sci..

[4] Zaidan I., Tavares L.P., Sugimoto M.A., Lima K.M., Negreiros-Lima G.L., Teixeira L.C., Miranda T.C., Valiate B.V., Cramer A., Vago J.P. (2022). Angiotensin-(1-7)/MasR axis promotes migration of monocytes/macrophages with a regulatory phenotype to perform phagocytosis and efferocytosis. JCI Insight.

[5] Vago J.P., Tavares L.P., Riccardi C., Teixeira M.M., Sousa L.P. (2020). Exploiting the pro-resolving actions of glucocorticoid-induced proteins Annexin A1 and GILZ in infectious diseases. Biomed. Pharmacother..

[6] Bereshchenko O., Migliorati G., Bruscoli S., Riccardi C. (2019). Glucocorticoid-induced leucine zipper (GILZ): A novel anti-inflammatory molecule. Front. Pharmacol..

[7] Vago J.P., Tavares L.P., Garcia C.C., Lima K.M., Perucci L.O., Vieira É.L., Nogueira C.R.C., Soriani F.M., Martins J.O., Silva P.M.R. (2015). The Role and Effects of Glucocorticoid-Induced Leucine Zipper in the Context of Inflammation RESOLUTION. J. Immunol..

[8] Vago J.P., Galvão I., Negreiros-Lima G.L., Teixeira L.C., Lima K.M., Sugimoto M.A., Moreira I.Z., Jones S.A., Lang T., Riccardi C. (2020). Glucocorticoid-induced leucine zipper modulates macrophage polarization and apoptotic cell clearance. Pharmacol. Res..

[9] Ellouze M., Vigouroux L., Tcherakian C., Woerther P.L., Guguin A., Robert O., Surenaud M., Tran T., Calmette J., Barbin T. (2020). Overexpression of GILZ in macrophages limits systemic inflammation while increasing bacterial clearance in sepsis in mice. Eur. J. Immunol..

[10] Ballegeer M., Vandewalle J., Eggermont M., Van Isterdael G., Dejager L., De Bus L., Decruyenaere J., Vandenbroucke R.E., Libert C. (2019). Overexpression of Gilz Protects Mice Against Lethal Septic Peritonitis. Shock.

[11] Souza J.A.M., Carvalho A.F.S., Grossi L.C., Zaidan I., de Oliveira L.C., Vago J.P., Cardoso C., Machado M.G., Souza G.V.S., Queiroz-Junior C.M. (2022). Glucocorticoid-Induced Leucine Zipper Alleviates Lung Inflammation and Enhances Bacterial Clearance during Pneumococcal Pneumonia. Cells.

[12] Ngo D., Beaulieu E., Gu R., Leaney A., Santos L., Fan H., Yang Y., Kao W., Xu J., Escriou V. (2013). Divergent Effects of Endogenous and Exogenous Glucocorticoid-Induced Leucine Zipper in Animal Models of Inflammation and Arthritis. Arthritis Rheum..

[13] Bruscoli S., Velardi E., Di Sante M., Bereshchenko O., Venanzi A., Coppo M., Berno V., Mameli M.G., Colella R., Cavaliere A. (2012). Long Glucocorticoid-induced Leucine Zipper (L-GILZ) Protein Interacts with Ras Protein Pathway and Contributes to Spermatogenesis Control. J. Biol. Chem..

[14] Romero Y., Vuandaba M., Suarez P., Grey C., Calvel P., Conne B., Pearce D., De Massy B., Hummler E., Nef S. (2012). The Glucocorticoid-Induced Leucine Zipper (GILZ) Is Essential for Spermatogonial Survival and Spermatogenesis. Sex. Dev..

[15] Cannarile L., Cuzzocrea S., Santucci L., Agostini M., Mazzon E., Esposito E., Muià C., Coppo M., di Paola R., Riccardi C. (2009). Glucocorticoid-Induced Leucine Zipper Is Protective in Th1-Mediated Models of Colitis. Gastroenterology.

[16] Gentili M., Hidalgo-Garcia L., Vezza T., Ricci E., Migliorati G., Rodriguez-Nogales A., Riccardi C., Galvez J., Ronchetti S. (2021). A recombinant glucocorticoid-induced leucine zipper protein ameliorates symptoms of dextran sulfate sodium-induced colitis by improving intestinal permeability. FASEB J..

[17] Negreiros-Lima G.L., Lima K.M., Moreira I.Z., Jardim B.L.O., Vago J.P., Galvão I., Teixeira L.C.R., Pinho V., Teixeira M.M., Sugimoto M.A. (2020). Cyclic AMP Regulates Key Features of Macrophages via PKA: Recruitment, Reprogramming and Efferocytosis. Cells.

[18] Vago J.P., Sugimoto M.A., Lima K.M., Negreiros-Lima G.L., Baik N., Teixeira M.M., Perretti M., Parmer R.J., Miles L.A., Sousa L.P. (2019). Plasminogen and the Plasminogen Receptor, Plg-RKT, Regulate Macrophage Phenotypic, and Functional Changes. Front. Immunol..

[19] Vago J.P., Nogueira C.R.C., Tavares L.P., Soriani F.M., Lopes F., Russo R.C., Pinho V., Teixeira M.M., Sousa L.P. (2012). Annexin A1 modulates natural and glucocorticoid-induced resolution of inflammation by enhancing neutrophil apoptosis. J. Leukoc. Biol..

[20] Vago J.P., Zaidan I., Perucci L.O., Brito L.F., Teixeira L.C., Silva C.M.S., Miranda T.C., Melo E.M., Bruno A.S., Queiroz-Junior C.M. (2023). Plasmin and plasminogen prevent sepsis severity by reducing neutrophil extracellular traps and systemic inflammation. J. Clin. Investig..

[21] Lima K.M., Vago J.P., Caux T.R., Negreiros-Lima G.L., Sugimoto M.A., Tavares L.P., Arribada R.G., Carmo A.A.F., Galvão I., Costa B.R.C. (2017). The resolution of acute inflammation induced by cyclic AMP is dependent on annexin A1. J. Biol. Chem..

[22] Kuziel W.A., Morgan S.J., Dawson T.C., Griffin S., Smithies O., Ley K., Maeda N. (1997). Severe reduction in leukocyte adhesion and monocyte extravasation in mice deficient in CC chemokine receptor 2. Proc. Natl. Acad. Sci. USA.

[23] Korns D.R., Frasch S.C., Fernandez-Boyanapalli R., Henson P.M., Bratton D.L. (2011). Modulation of Macrophage Efferocytosis in Inflammation. Front. Immunol..

[24] Degboé Y., Rauwel B., Baron M., Boyer J.F., Ruyssen-Witrand A., Constantin A., Davignon J.L. (2019). Polarization of rheumatoid macrophages by TNF targeting through an IL-10/STAT3 mechanism. Front. Immunol..

[25] Mu X., Shi W., Xu Y., Xu C., Zhao T., Geng B., Yang J., Pan J., Hu S., Zhang C. (2018). Tumor-derived lactate induces M2 macrophage polarization via the activation of the ERK/STAT3 signaling pathway in breast cancer. Cell Cycle.

[26] Shiraishi D., Fujiwara Y., Komohara Y., Mizuta H., Takeya M. (2012). Glucagon-like peptide-1 (GLP-1) induces M2 polarization of human macrophages via STAT3 activation. Biochem. Biophys. Res. Commun..

[27] Yao R.R., Li J.H., Zhang R., Chen R.X., Wang Y.H. (2018). M2-polarized tumor-associated macrophages facilitated migration and epithelial-mesenchymal transition of HCC cells via the TLR4/STAT3 signaling pathway. World J. Surg. Oncol..

[28] Chiang N., Fredman G., Bäckhed F., Oh S.F., Vickery T., Schmidt B.A., Serhan C.N. (2012). Infection regulates pro-resolving mediators that lower antibiotic requirements. Nature.

[29] Nathan C. (2002). Points of control in inflammation. Nature.

[30] Sugimoto M.A., Sousa L.P., Pinho V., Perretti M., Teixeira M.M. (2016). Resolution of inflammation: What controls its onset?. Front. Immunol..

[31] Buckley C.D., Gilroy D.W., Serhan C.N., Stockinger B., Tak P.P. (2013). The resolution of inflammation. Nat. Rev. Immunol..

[32] Sugimoto M.A., Vago J.P., Perretti M., Teixeira M.M. (2019). Mediators of the Resolution of the Inflammatory Response. Trends Immunol..

[33] Perucci L.O., Sugimoto M.A., Gomes K.B., Dusse L.M., Teixeira M.M., Sousa L.P. (2017). Annexin A1 and specialized proresolving lipid mediators: Promoting resolution as a therapeutic strategy in human inflammatory diseases. Expert Opin. Ther. Targets.

[34] Carmo A.A., Costa B.R., Vago J.P., de Oliveira L.C., Tavares L.P., Nogueira C.R., Ribeiro A.L.C., Garcia C.C., Barbosa A.S., Brasil B.S. (2014). Plasmin induces in vivo monocyte recruitment through Protease-Activated Receptor-1–, MEK/ERK-, and CCR2-Mediated Signaling. J. Immunol..

[35] Sugimoto M.A., Ribeiro A.L.C., Costa B.R.C., Vago J.P., Lima K.M., Carneiro F.S., Ortiz M.M.O., Lima G.L.N., Carmo A.A.F., Rocha R.M. (2017). Plasmin and plasminogen induce macrophage reprogramming and regulate key steps of inflammation resolution via annexin A1. Blood.

[36] Galvão I., Vago J.P., Barroso L.C., Tavares L.P., Queiroz-Junior C.M., Costa V.V., Carneiro F.S., Ferreira T.P., Silva P.M.R., Amaral F.A. (2017). Annexin A1 promotes timely resolution of inflammation in murine gout. Eur. J. Immunol..

[37] Sugimoto M.A., Vago J.P., Teixeira M.M., Sousa L.P. (2016). Annexin A1 and the Resolution of Inflammation: Modulation of Neutrophil Recruitment, Apoptosis, and Clearance. J. Immunol. Res..

[38] De Carvalho Santuchi M., Dutra M.F., Vago J.P., Lima K.M., Galvão I., De Souza-Neto F.P., Silva M.M.E., Oliveira A.C., De Oliveira F.C.B., Gonçalves R. (2019). Angiotensin-(1-7) and Alamandine Promote Anti-inflammatory Response in Macrophages In Vitro and In Vivo. Mediat. Inflamm..

[39] Beaulieu E., Morand E. (2011). Role of GILZ in immune regulation, glucocorticoid actions and rheumatoid arthritis. Nat. Rev. Rheumatol..

[40] Bruscoli S., Febo M., Riccardi C., Migliorati G. (2021). Glucocorticoid Therapy in Inflammatory Bowel Disease: Mechanisms and Clinical Practice. Front. Immunol..

[41] Hoppstädter J., Kessler S.M., Bruscoli S., Huwer H., Riccardi C., Kiemer A.K. (2015). Glucocorticoid-Induced Leucine Zipper: A Critical Factor in Macrophage Endotoxin Tolerance. J. Immunol..

[42] Ricci E., Ronchetti S., Gabrielli E., Pericolini E., Gentili M., Roselletti E., Vecchiarelli A., Riccardi C. (2018). GILZ restrains neutrophil activation by inhibiting the MAPK pathway. J. Leukoc. Biol..

[43] Espinasse M.-A., Pépin A., Virault-Rocroy P., Szely N., Chollet-Martin S., Pallardy M., Biola-Vidamment A. (2015). Glucocorticoid-Induced Leucine Zipper Is Expressed in Human Neutrophils and Promotes Apoptosis through Mcl-1 Down-Regulation. J. Innate Immun..

[44] Roca H., Varsos Z.S., Sud S., Craig M.J., Ying C., Pienta K.J. (2009). CCL2 and Interleukin-6 Promote Survival of Human CD11b+ Peripheral Blood Mononuclear Cells and Induce M2-type Macrophage Polarization. J. Biol. Chem..

[45] Sierra-Filardi E., Nieto C., Domínguez-Soto Á., Barroso R., Sánchez-Mateos P., Puig-Kroger A., López-Bravo M., Joven J., Ardavín C., Rodríguez-Fernández J.L. (2014). CCL2 shapes macrophage polarization by GM-CSF and M-CSF: Identification of CCL2/CCR2-dependent gene expression profile. J. Immunol..

[46] Dalli J., Serhan C.N. (2017). Pro-Resolving Mediators in Regulating and Conferring Macrophage Function. Front. Immunol..

[47] Daly C., Rollins B.J. (2003). Monocyte chemoattractant protein-1 (CCL2) in inflammatory disease and adaptive immunity: Therapeutic opportunities and controversies. Microcirculation.

[48] Tanaka T., Terada M., Ariyoshi K., Morimoto K. (2010). Monocyte chemoattractant protein-1/CC chemokine ligand 2 enhances apoptotic cell removal by macrophages through Rac1 activation. Biochem. Biophys. Res. Commun..

[49] Ross E.A., Devitt A., Johnson J.R. (2021). Macrophages: The good, the bad, and the gluttony. Front. Immunol..

[50] Mosser D.M. (2003). The many faces of macrophage activation. J. Leukoc. Biol..

[51] Mosser D.M., Edwards J.P. (2008). Exploring the full spectrum of macrophage activation. Nat. Rev. Immunol..

[52] Shapouri-Moghaddam A., Mohammadian S., Vazini H., Taghadosi M., Esmaeili S.A., Mardani F., Seifi B., Mohammadi A., Afshari J.T., Sahebkar A. (2018). Macrophage plasticity, polarization, and function in health and disease. J. Cell. Physiol..

[53] A Fadok V., Bratton D.L., Konowal A., Freed P.W., Westcott J.Y., Henson P.M. (1998). Macrophages that have ingested apoptotic cells in vitro inhibit proinflammatory cytokine production through autocrine/paracrine mechanisms involving TGF-beta, PGE2, and PAF. J. Clin. Investig..

[54] Kawakubo A., Miyagi M., Yokozeki Y., Nakawaki M., Takano S., Satoh M., Itakura M., Inoue G., Takaso M., Uchida K. (2022). Origin of M2 Mϕ and its macrophage polarization by TGF-β in a mice intervertebral injury model. Int. J. Immunopathol. Pharmacol..

[55] Raes G., Van den Bergh R., De Baetselier P., Ghassabeh G.H., Scotton C., Locati M., Mantovani A., Sozzani S. (2005). Arginase-1 and Ym1 Are Markers for Murine, but Not Human, Alternatively Activated Myeloid Cells. J. Immunol..

[56] Proto J.D., Doran A.C., Gusarova G., Yurdagul A., Sozen E., Subramanian M., Islam M.N., Rymond C.C., Du J., Hook J. (2018). Regulatory T Cells Promote Macrophage Efferocytosis during Inflammation Resolution. Immunity.

[57] Schif-Zuck S., Gross N., Assi S., Rostoker R., Serhan C.N., Ariel A. (2010). Saturated-efferocytosis generates pro-resolving CD11blow macrophages: Modulation by resolvins and glucocorticoids. Eur. J. Immunol..

[58] Watanabe S., Alexander M., Misharin A.V., Budinger G.S. (2019). The role of macrophages in the resolution of inflammation. J. Clin. Investig..

[59] Ariel A., Serhan C.N. (2012). New Lives Given by Cell Death: Macrophage Differentiation Following Their Encounter with Apoptotic Leukocytes during the Resolution of Inflammation. Front. Immunol..

[60] Byrne A., Reen D.J. (2002). Lipopolysaccharide Induces Rapid Production of IL-10 by Monocytes in the Presence of Apoptotic Neutrophils. J. Immunol..

[61] Pello O.M. (2016). Macrophages and c-Myc cross paths. Oncoimmunology.

[62] Berrebi D., Bruscoli S., Cohen N., Foussat A., Migliorati G., Bouchet-Delbos L., Maillot M.-C., Portier A., Couderc J., Galanaud P. (2003). Synthesis of glucocorticoid-induced leucine zipper (GILZ) by macrophages: An anti-inflammatory and immunosuppressive mechanism shared by glucocorticoids and IL-10. Blood.

[63] Maciuszek M., Rydz L., Świtakowska I., Kemenade B.L.V.-V., Chadzińska M. (2019). Effects of stress and cortisol on the polarization of carp macrophages. Fish Shellfish. Immunol..

[64] Bruscoli S., Riccardi C., Ronchetti S. (2021). GILZ as a Regulator of Cell Fate and Inflammation. Cells.

[65] Labonte A.C., Tosello-Trampont A.-C., Hahn Y.S. (2014). The Role of Macrophage Polarization in Infectious and Inflammatory Diseases. Mol. Cells.

[66] Wang N., Liang H., Zen K. (2014). Molecular mechanisms that influence the macrophage M1–M2 polarization balance. Front. Immunol..

[67] Bruscoli S., Sorcini D., Flamini S., Gagliardi A., Adamo F., Ronchetti S., Migliorati G., Bereshchenko O., Riccardi C. (2018). Glucocorticoid-Induced Leucine Zipper Inhibits Interferon-Gamma Production in B Cells and Suppresses Colitis in Mice. Front. Immunol..

[68] Beaulieu E., Ngo D., Santos L., Yang Y.H., Smith M., Jorgensen C., Escriou V., Scherman D., Courties G., Apparailly F. (2010). Glucocorticoid-induced leucine zipper is an endogenous antiinflammatory mediator in arthritis. Arthritis Rheum..

[69] Baban B., Marchetti C., Khodadadi H., Malik A., Emami G., Lin P.-C., Arbab A.S., Riccardi C., Mozaffari M.S. (2018). Glucocorticoid-Induced Leucine Zipper Promotes Neutrophil and T-Cell Polarization with Protective Effects in Acute Kidney Injury. J. Pharmacol. Exp. Ther..

[70] Pinheiro I., Dejager L., Petta I., Vandevyver S., Puimège L., Mahieu T., Ballegeer M., Van Hauwermeiren F., Riccardi C., Vuylsteke M. (2013). LPS resistance of SPRET/Ei mice is mediated by Gilz, encoded by the *Tsc22d3* gene on the X chromosome. EMBO Mol. Med..

[71] Hoppstädter J., Diesel B., Linnenberger R., Hachenthal N., Flamini S., Minet M., Leidinger P., Backes C., Grässer F., Meese E. (2019). Amplified Host Defense by Toll-Like Receptor-Mediated Downregulation of the Glucocorticoid-Induced Leucine Zipper (GILZ) in Macrophages. Front. Immunol..

