# Supplementary material for: GILZ Modulates the Recruitment of Monocytes/Macrophages Endowed with a Resolving Phenotype and Favors Resolution of Escherichia coli Infection
Source: Cells. 2023 May 17;12(10):1403. doi: 10.3390/cells12101403 (PMC10217061; doi:10.3390/cells12101403)
Supplement: Supplementary file 1 [file cells-12-01403-s001.zip › cells-2376724-supplementary.pdf]

Supplementary material

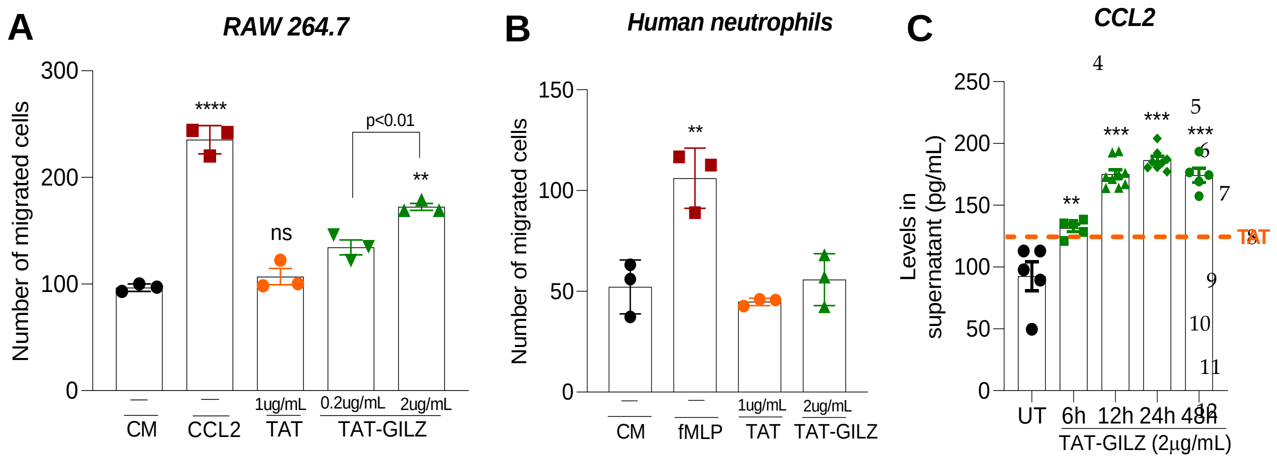

Supplementary Figure S1. TAT-GILZ induces macrophage migration and CCL2 production *in vitro*. (**A**) RAW 264.7 cells were incubated for 4 h and allowed to migrate through polycarbonate membranes (pore size 5 µm) using TAT (1 µg/ml) or TAT-GILZ at different concentrations at the lower chamber (0.2 and 2 µg/ml). The CCL2 chemokine (100 ng/ml) was used as a positive chemoattractant control for macrophages. (**B**) Human neutrophils were isolated from human peripheral blood and then allowed to migrate on polycarbonate membranes (pore size 3 µm) for 4 h, using TAT (1 µg/ml), TAT-GILZ (2 µg/mL) or fMLP (10^{-9}M - positive chemoattractant control for neutrophils). (**C**) Levels of CCL2 in BMDMs supernatants at different time points upon TAT-GILZ treatment. ** Denotes p<0.01, *** p<0.001, ****p<0.0001 when comparing to the CM (control medium) or untreted cells, by one-way ANOVA. Statistical diference among the grups are depicted in the figure A. Results in A and B are expressed as the number of migrated cells counted in five random fields using light microscope, after membrane staining and are presented as mean ± SEM. A and C are representative results of two independent experiments performed in biological triplicates (*n* = 3) or quintuplicates (C). Graphs B represent data collected from different healthy volunteers (*n* =3).

In vivo

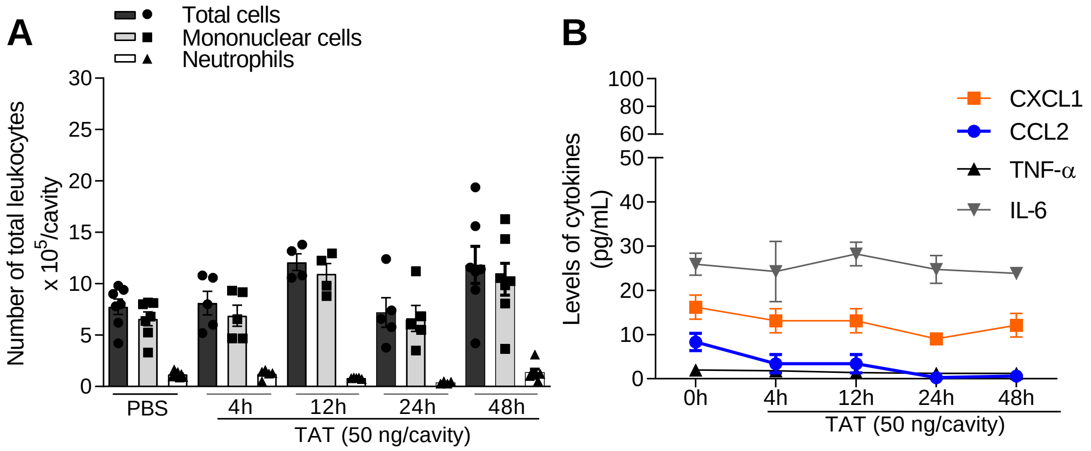

Supplementary Figure S2. Effects of TAT injection into the pleural cavity of mice. (**A)** Represents the couting of leukocyte into the pleural cavity of C57BL/6 mice after TAT injection (50 ng). (**B**) Inflammatory cytokines and chemokines were measured by ELISA in cell free pleural exudates at different time points. Results are presented as mean ± SEM of 5-7 mice per group.
